# Study of photosynthetic pigments changes of maize (*Zea mays* L.) under nano Tio_2_ spraying at various growth stages

**DOI:** 10.1186/2193-1801-2-247

**Published:** 2013-05-30

**Authors:** Elham Morteza, Payam Moaveni, Hossein Aliabadi Farahani, Mohammad Kiyani

**Affiliations:** Agronomy and Plant Breeding Department, Abureyhan Campus University of Tehran, Emam Reza Boulevard, PO. Box:11365/4117, Pakdasht, Tehran, Iran; Young Researchers Club, Islamic Azad University, Shahr-e-Qods Branch Fath Highway, Shahid Kalhor Boulevard, PO. Box: 37515-374, Shahr-e-Qods, Tehran, Iran; Islamic Azad University, Shahr-e-Qods Branch, Tehran, Iran; Islamic Azad University, Tabriz Branch, Vali Asr Alley, PO. Box: 1655, Tabriz, Iran

**Keywords:** Anthocyanins, Carotenoids, Chlorophyll, Maize (*Zea mays* L.), Nano Tio_2_

## Abstract

Tests were done on the effects of treatments of titanium dioxide spray on corn (*Zea mays L*.). The study was conducted as a factorial experiment in a randomized complete block design with four replications. Treatments consisted of two factors; the first factor was stage of plant growth that spraying was applied (vegetative stage, appearance of male flowers and female flowers); and the second factor was that of different concentrations of titanium dioxide nanoparticles (Tio_2_) that consisted of spray with water (control), titanium dioxide or bulk, nano titanium dioxide at concentrations of 0.01% and 0.03%. Results showed that effect of nano Tio_2_ was significant on chlorophyll content (a and b), total chlorophyll (a + b), chlorophyll a/b, carotenoids and anthocyanins. The maximum amount of pigment was recorded from the treatment of nano Tio_2_ spray at the reproductive stage (appearance of male and female flowers) in comparison with control. Thus, an application of nanoparticles (nanao Tio_2_) can facilitate an increase in crop yield, especially corn yield.

## Introduction

Corn (*Zea mays* L.) is an important, strategic cereal crop that has a relatively short growing period and produces a high yield. Corn ranks third in world in terms of production after wheat and rice (Raji 2003). Nanoparticles, which are 1–100 nm in diameter (Ruffini Castiglione and Cremonini 2009) have a large specific surface area that can provides for a good level of reactivity and this character facilitates effective absorption of fertilizers and pesticides at nanoscale (Sheykhbaglou et al., 2010). Thus nano particles can be used to increase the supply of elements to plant shoots and foliage. Nano particles appropriate for such application are those of are nano Tio_2,_ nano zinc, nano iron, nano aluminum and nano silver (Reynolds 2002). Application of nanoparticles can also increase seed germination and seedling growth. Furthermore nanoparticles can facilitate enhanced ability of water and fertilizer absorption by roots, and increase antioxidant enzyme activity such as superoxide dismutase and catalase. Thus, nanoparticles can increase plant resistance against different stresses (Harrison 1996). Many studies have reported that nano particles have favorable effects on plant growth and development. Titanium dioxide nanoparticles are used in agriculture to increase growth and can improve yield by approximately 30%; improve the rate of photosynthesis and reduce disease (Chao and Choi 2005). Titanium dioxide is used commercially as the most appropriate catalyst for photo catalytic reactions; upon exposure to ultraviolet light it mineralizes the organic chemicals in rivers to water and carbon dioxide with the potential to destroy microorganisms (Owolade et al., 2008). Lei et al., ( 2007) found that, nano Tio_2_ increases photosynthesis and plant growth of spinach and enhances absorption and transmission of the sun’s energy to electron energy and activates chemical energy. Other research has reported that nano Tio_2_, could greatly improve whole chain electron transport, photo reduction activity of photosystem II, O2-evolving and photophosphorylation activity of spinach Chl, not only under visible light, but also energy-enriched electron from nanoanatase Tio_2_, which entered the Chl under ultraviolet light and was transferred in a photosynthetic electron transport chain and reduced NADP + be into NADPH, and coupled to photophosphorylation and stimulated the transformation of electron energy to ATP. In another experiment, Zheng et al. ( 2005), considered, germination rate and vigor index of spinach old seeds that treated with the nano Tio_2_ (concentrations of 0, 0.25, 0.5, 1, 1.5, 2, 2.5, 4, 6 and 8 percent) and Tio_2_ (concentrations of 0, 0.25, 0.5, 1, 1.5, 2, 2.5, 4, 6 and 8 percent) and observed that evaluations for these traits increased in seeds treated with concentrations of 0.25 to 4 percent of nano Tio2 in comparison with Tio_2_. Evaluations for chlorophyll levels, ribulose 1,5-bisphosphate carboxylase activity, dry weight and fresh weight in seedlings that had been treated with 2.5 percent were higher than evaluations for treatments at other concentrations of nano Tio_2_ and Tio_2_ (bulk). Lu et al., ( 2002) reported that evaluations for nitrate reductase, super oxide dismutase and catalase increased in germinated soybean seeds that had been treated with nano Tio_2_, and that levels of water water and fertilizer use efficiency increased. The research results of Moaveni et al., ( 2011) on effects of different nano titanium dioxide concentrations (0.01, 0.02, 0.03 percent) and titanium dioxide (bulk) spray treatment on barley plants showed that traits of grain yield, number of ears and harvest index in all treatments of nano titanium dioxide application were more effective than the control treatment. It was demonstrated that barley plants sprayed with nano titanium dioxide at concentrations of 0.03% was 21.32% produced higher evaluations for grain yield compared to the control. Also, Owolade et al., ( 2008) concluded that effects of nano Tio_2_ spray on evaluations for traits of seed number per pod, 1000 seed weight, grain yield, leaf area, pod number per plant and pod length of *Vina unguiculata*, were significant and evaluations of these traits significantly increased compared to the control. So, this study was done to examine changes of photosynthetic pigments in maize (*Zea mays* L.) sprayed with nano Tio_2_ at various stages of growth and development.

## Material and methods

This study was done as a factorial experiment in a complete randomized block design. Treatments consisted of two factors. The first factor tested spraying application at various stages of plant development (vegetative stage, appearance of male flowers and female flowers) and the second factor tested different amounts of titanium dioxide nanoparticles (control or sprayed with water, titanium dioxide or bulk and nano titanium dioxide with the concentration of 0.01% and 0.03%. Maize seeds were planted within lysimeters in July 2011. Fertilization and plant feeding was done according to recommendations from results of a soil test. Spraying treatment was based on growth stages and concentrations of nanoTio_2._ Evaluations were made for the characters of chlorophyll, total chlorophyll, carotenoids and anthocyanins. The amounts of chlorophyll (a, b, a/b and total) and carotenoids were measured in accordance with the method cited in Lichtenthaler ( 1987) and evaluation of the level of anthocyanins was measured in accordance with the method of Wanger ( 1979). Finally, evaluations for traits were determined by the following formula;

Data were subjected to analysis of variance (ANOVA) using Statistical Analysis System (SAS Institute 1988) and followed by Duncan’s multiple range tests. Terms were considered significant at P ≤ 0.01.

## Results

Results of evaluations of plant characters showed that the effect of spraying time was significant (P ≤ 0.01, Table 1) on all traits with the exception of t chlorophyll a/b. The effect of different amounts of titanium dioxide nanoparticles (Tio_2_) was significant on all traits (P ≤ 0.01, Table 1).

**Table 1:** **Results of variance analysis of the Zea mays treated with nano Tio**_**2**_**at vegetative and reproductive stages**

Means square							
Anthocyanins	Carotenoids	Chlorophyll a/b	Chlorophyll a + b	Chlorophyll b	Chlorophyll a	df	Sources of variation
15.38**	0.0007 ^ns^	0.0006**	0.005**	0.00369**	0.00053 ^ns^	3	Replication
47.27**	0.005**	0.0001 ^ns^	0.039**	0.00615**	0.01350**	2	(a) Times of spraying
192.66**	0.005**	0.0026**	0.554**	0.01722**	0.07128**	3	(b) Amounts of Tio_2_
1.10^ns^	0.0007**	0.0001 ^ns^	0.001**	0.00005^ns^	0.00116**	6	b × a
2.88	0.0001	0.00008	0.0005	0.00017	0.00018	33	Error

Note: Ns, Non Significant, * and **, Significant at 5% and 1% levels respectively.

The effect of different amounts and times of titanium dioxide nanoparticles (Tio_2_) applications, was significant, on traits of chlorophyll a, chlorophyll a + b, and carotenoids (P ≤ 0.01, Table 1).

According to Tables 2, 3 and 4, the highest amount of chlorophyll a, b and (a + b), was obtained by spraying of nano titanium dioxide at the concentration of 0.03% at the stage of the appearance of male and female flowers and the lowest evaluation for this traits was achieved by spraying with distilled water (control treatment) at the vegetative stage.

**Table 2:** **Means comparsion of nano Tio**_**2**_**spraying times on traits of Zea mays**

Anthocyanins (μmol.g^-1^.fw)	Carotenoids (mg.g^-1^.fw)	Chlorophyll a/b	Total chlorophyll (a + b) (mg.g^-1^.fw)	Chlorophyll b (mg.g^-1^.fw)	Chlorophyll a (mg.g^-1^.fw)	Times of spraying
31.12c	1.61c	1.25a	4.79b	2.12b	2.67b	Vegetative stage
32.82b	1.64b	1.26a	4.87a	2.15a	2.72a	Appearance of male flowers
34.56 a	1.65a	1.25a	4.88a	2.15a	2.72a	Appearance of female flowers

Note: Means in the same columns and rows, followed by the same letter are not significantly difference (P < 0.01).

**Table 3:** **Means comparison of nano on traits of Zea mays**

Anthocyanins (μmol.g^-1^.fw)	Carotenoids (mg.g^-1^.fw)	Chlorophyll a/b	Total chlorophyll (a + b) (mg.g^-1^.fw)	Chlorophyll b (mg.g^-1^.fw)	Chlorophyll a (mg.g^-1^.fw)	Amounts of nanoTio_2_
27d	1.60b	1.24c	4.73d	2.10c	2.63 d	(Distilled water) Control
32c	1.64a	1.25bc	4.77c	2.11c	2.65 c	(Bulk) Tio_2_
34b	1.65a	1.26b	4.89b	2.16b	2.73b	(0.01%) Nano Tio_2_
37a	1.64a	1.27a	4.98a	2.18a	2.80 a	(0.03%) Nano Tio_2_

Note: Means in the same columns and rows, followed by the same letter are not significantly difference (P < 0.01).

**Table 4:** **Means comparison of spraying of times and amounts of nano Tio**_**2**_**on traits of Zea mays**

Carotenoids (mg.g^-1^.fw)	Total chlorophyll (a + b) (mg.g^-1^.fw)	Chlorophyll a (mg.g^-1^.fw)	Amounts of nanoTio_2_	Times of spraying
1.56f	4.70f	2.61f	(Distilled water) Control	Vegetative stage
1.62e	4.72ef	2.63ef	(Bulk) Tio_2_	Vegetative stage
1.64ed	4.83c	2.69c	(0.01%) Nano Tio_2_	Vegetative stage
1.64ed	4.90b	2.74b	(0.03%) Nano Tio_2_	Vegetative stage
1.63ed	4.75e	2.63ef	(Distilled water) Control	Appearance of male flowers
1.65abc	4.80dc	2.67cd	(Bulk) Tio_2_	Appearance of male flowers
1.65abc	b4.93b	2.75b	0.01% Nano Tio_2_	Appearance of male flowers
1.63edc	5.01a	2.82a	0.03% Nano Tio_2_	Appearance of male flowers
1.63ed	4.76de	2.64e	(Distilled water) Control	Appearance of female flowers
1.65abc	4.79dc	2.66d	(Bulk) Tio_2_	Appearance of female flowers
1.66a	4.92b	2.74b	()0.01% Nano Tio_2_	Appearance of female flowers
1.66a	5.04a	2.84a	(0.03%) Nano Tio_2_	Appearance of female flowers

Note: Means in the same columns and rows, followed by the same letter are not significantly difference (P < 0.01).

Also, the highest amount of chlorophyll a b was gained by spraying nano titanium dioxide at the concentration of 0.03% at the stage of the appearance of male flowers and vegetative stage and it higher than titanium dioxide (bulk) and control treatment. The highest amount of cartenoids was obtained by the use of nano titanium dioxide at the stage of the appearance of female flowers. But effects of nano titanium dioxide application at concentrations of 0.03% and 0.01% and titanium dioxide (bulk) were not significantly different and have the highst amounts of cartenoids in comparison with the control.

According to these results, spraying at the stage of the appearance female flowers, and male flowers and vegetative stage, respectively had the lowest evaluations for antocyanin. It should be noted that, the use of the nano titanium dioxide at the concentrations of 0.03% and 0.01%, titanium dioxide (bulk), and the control had the lowest evaluations for antocyanin respectively.

## Discussion

Our study results indicate that nano Tio_2_ plays a significant role on increasing of pigments *Zea mays*, so that higher amounts of pigments obtained with spraying of nano Tio_2_ in reproductive Stages of plant (Appearance of male and female flowers) in compared with sparaying tio_2_ (bulk) and distilled water (control) sparaying in vegetative stage because of, remobilization of photosynthetic material from leaves to fruits at flowering time (Appearance of male and female flowers), leaves are starting to aging and the first synthetic symptom of aging leaves, is that the structure and function of chloroplasts was changed, and a vital factor is the change occurring in the membrane system of chloroplasts. integration of the chloroplast envelope, especially the inner envelope, played a positive role in many aspects. For instance, during the course of leaves coming back green, reformation of the thylakoid membranes might come from the chloroplast inner envelope (Woolhouse 1984). In contrast, if chloroplast membranous system suffers destruction, it will influence the regular physiological function and metabolism that might cause chloroplast aging. In our experiment, probably membrane permeability of aging leaves in Appearance of male and female flowers that sprayed with nano Tio_2,_ was slowly rose. The results showed that nano Tio_2_ could stabilize the integrality of chloroplast membrane and protect the chloroplasts from aging in flowering times in contrast with control. Therefore with nano Tio_2_ treatment spraying, content of chlorophyll (a and b), was higher than the control. the amount of chlorophyll in the reproductive stage was even more vegetative time. In fact, titanium dioxide (nano) can improve structure of chlorophyll and better capture of sunlight, can facilitate manufacture of pigments and transformation of light energy to active electron and chemical activity and increases photosynthetic efficiency, stimulates rubisco activase and also increases photosynthesis. The studies on improving photosynthesis of spinach suggested that nano-anatase Tio_2_ could increase light absorbance, accelerate transport and transformation of the light energy, protect chloroplasts from ageing and prolong photosynthetic time of chloroplasts (Yang and Hong. 2006). However, bulk Tio_2_ effects on pigments was not as significant as nano Tio_2_, as the grain size of nano Tio_2_ is much smaller than that of bulk TiO_2_, which entered spinach cell more easily. These results are in accordance with those reported by Lei et al. ( 2007) and Zheng et al. ( 2005), so that Gao et al. ( 2008) in a study on the effects of different concentrations of nano titanium dioxide on the spinach traits, concluded that, chlorophyll amounts in treatment with nano Tio_2_ showed significant increase, and it was 17 times of control amount, also photosyntetic rate amount showed 29% enhancement in compared with the control. In a study, nano Tio_2_ effects on nitrogen metabolism in spinach improved spinach growth and increased protein and chlorophyll contents sensibly (Yang and Hong. 2006). As a result of this experiment, the use of nano titanium dioxide at 0.03% concentration in the appearance of flowers stage, increased chlorophyll amounts (total, a, b) and anthocyanin. Application of nano titanium dioxide at 0.01%, 0.03% concentrations and titanium dioxide (bulk) in the appearance of female flowers, increased the amount of carotenoids of corn.

Nano Tio_2_ probably affected on photoreduction activities of photosystem II and electron transport chain and so increased amount of pigments. suggested that photosynthesis promoted by nano Tio_2_ might be related to activation of photochemical reaction of crop chloroplasts. Thus, according to the results of this study, use of nanoparticles (nanao Tio_2_) would be used to increase of *zea mays* yield, because of increase of pigment amounts (Yang and Hong. 2006).

## References

[CR1] Chao SHL, Choi HS (2005). Method for providing enhanced photosynthesis.

[CR2] Gao F, Hong F, Liu C, Zheng L, Su M, Wu X, Yang F, Wu C, Yang P (2008). Mechanism of nano. anatase Tio2 on promoting photosynthetic carbon reaction of spinach. Biol Trace Elem Res J.

[CR3] Harrison CC (1996). Evidence for intramineral macromolecules containing protein from plant silicas. Phytochem J.

[CR4] Lei Z, Mingyu S, Chao L, Liang C, Hao H, Xiao W, Xiaoqing L, Fan Y, Fengqing G, Fashui H (2007). Effects of nanonatase Tio2 on photosynthesis of spinach chloroplasts under different light illumination. Biol Trace Elem Res J.

[CR5] Lichtenthaler HK (1987). Chlorophylls and carotenoids: Pigments of photosynthetic biomembranes. Methods Enzymol.

[CR6] Lu CM, Zhang CY, Wen JQ, Wu GR (2002). Effects of nano material on germination and growth of soybean. Soybean Sci.

[CR7] Moaveni P, Talebi A, Farahani A, Maroufi H, Maroufi K (2011). Study of Tio2 nano particles spraying effect on the some physiological prameters in barley (*Hordem Vulgare* L.). Adv Environ Biol.

[CR8] Owolade OF, Ogunleti DO, Adenekan MO (2008). Titanum dioxide affects diseases, development and yield of edibile cowpea. EJEAFChe.

[CR9] Raji JA (2003). Inter. cropping soybean and maize in a derived savanna echology. Afr J Biotechnol.

[CR10] Reynolds GH (2002). Forward to the future nanotechnology and the regulatory policy. Pac Res Inst.

[CR11] Ruffini Castiglione M, Cremonini R (2009). Nanoparticles and higher plants. Caryologia.

[CR12] (1988). Statistics analysis system user’s guide: statistics.

[CR13] Sheykhbaglou R, Sedghi M, Tajbakhsh Shishvan M, Seyed Sharifi R (2010). Effect of nano. iron particles on agronomic traits of soybean. Not Sci Biol.

[CR14] Wanger GJ (1979). Content and vacuole/extra vacuole distribution of neutral sugars, free amino acids, and anthocyanis in protoplasts. Plant Physiol.

[CR15] Woolhouse HW (1984). The biochemistry and regulation of senescence in chloroplasts. Can. J. Bot.

[CR16] Yang F, Hong FS (2006). Influence of nano-anatase Tio2 on the nitrogen metabolism of growing spinach. Biol Trace Element Res.

[CR17] Zheng L, Hong F, Lu S, Liu C (2005). Effect of nano. Tio2 on strength of naturally aged seeds and growth of spinach. Biol Trace Elem Res J.

